# Pulmonary vein anatomy variants as a biomarker of atrial fibrillation – CT angiography evaluation

**DOI:** 10.1186/s12872-018-0884-3

**Published:** 2018-07-13

**Authors:** M. Skowerski, I. Wozniak-Skowerska, A. Hoffmann, S. Nowak, T. Skowerski, M. Sosnowski, A. M. Wnuk-Wojnar, K. Mizia-Stec

**Affiliations:** 1 First Department of Cardiology, School of Medicine in Katowice, Medical University of Silesia, Katowice, Poland; 2 Department of Cardiology, School of Health Sciences, Medical University of Silesia, Katowice, Poland; 3 Unit of Noninvasive Cardiovascular Diagnostics, Medical University of Silesia, Katowice, Poland

**Keywords:** Atrial fibrillation, Pulmonary vein isolation, CT angiography

## Abstract

**Background:**

It has been suggested that changes in pulmonary veins (PV) and left atrium (LA) anatomy may have an influence on initiating atrial fibrillation (AF) and the effectiveness of pulmonary vein isolation (PVI) in patients (pts) with atrial fibrillation.

The aim of the study was to assess anatomy abnormalities of the PV and LA in the patients with the history of AF and compare it with the control group(CG).

**Methods:**

The multi-slice tomography (MSCT) scans were performed in 224 AF pts. before PVI (129 males, mean age 59 ± 9 yrs). The CG consisted of 40 pts. without AF (26 males, age 45 ± 9 yrs). LA and PV anatomy were evaluated. Diameters of PV ostia were measured in two directions: anterior-posterior (AP) and superior-inferior (SI) automatically using Vitrea 4.0.

**Results:**

Pulmonary veins anatomy variants were observed more frequently in the atrial fibrillation group - 83 pts. (37%) vs 6 pts. (15%) in CG; 9% (21 pts) left common ostia (CO), 2% (5 pts) right CO, 19% (42 pts) additional right PV (APV), (1.8%) 4 pts. APV left, 8% right early branching (EB) and 3.5% left EB. The LA diameter differed significantly in AF vs CG group (41.2 ± 6 mm vs 35 ± 4.2 mm, *p* < 0.0001) respectively.

**Conclusions:**

The anomalies of pulmonary vein anatomy occurred more often in pts. with AF. They can be defined as an image biomarkers of atrial fibrillation. Right additional (middle) pulmonary vein was the most important anomaly detected in AF patients as well as enlargered diameters of the LA and PV ostia.

## Background

Atrial fibrillation (AF) is a frequently occurring arrhythmia that impairs the life functioning, is associated with the increasing medical supervision, the risk of other diseases and pharmacological therapy complications. Pulmonary vein isolation (PVI) over the last decade has become the most demanded method for AF treatment. Even with experience in ablation, knowledge of left atrial anatomy and pulmonary vein anatomy is necessary [1]. A detailed visualization of the left atrium (LA) and pulmonary vein (PV) anatomy can be obtained by several different imaging methods including transesophageal or intracardiac echocardiography, rotational angiography, multislice computed tomography (MSCT) or three-dimensional gadolin-enhanced magnetic resonance. The MSCT is one of the most common and objective methods [2–5]. Anatomic varations of left atrium such as common ostia, additional pulmonary vein or early branching are common and previously described.

Some authors suggest that those anomalies may play a significant role in the pathophysiology of atrial fibrillation and even increase its prevalence [4, 6]. The safety and effectiveness of PVI in patients with AF is still under intensive clinical investigation [5, 7]. Therefore, the purpose of this study was to evaluate the occurrence of anatomy anomalies of PV and the left atrium using MSCT scans performed in patients with AF and compare the results with no history of AF (control group).

## Methods

### Study population

A total of 271 pts. are analyzed in this paper. The study population (AF group) consisted of 224 patients (129 males, mean age 59 ± 9 yrs., range 22–74 yrs) with the history of paroxysmal and/or persistent symptomatic non-valvular AF referred to our hospital for qualification for PVI. The study was approved by the ethics committee and conformed to the Declaration of Helsinki. An informed written consent was obtained from every patient enrolled in the study. All the patients from the study group had well documented episodes of AF using Holter ECG and/or surface ECG. MSCT images were performed for LA and PV visualization. Exclusion criteria involved: history of congenital heart diseases, left ventricular systolic dysfunction (LV EF < 35%), hemodynamically significant valvular heart diseases. The subjects who underwent PVI were also excluded from the study.

The control group (CG) consisted of 40 pts. (26 males, mean age 45 ± 9 yrs) with no history of AF. MSCT scans were performed due to the symptoms of suspected pathologies in a coronary artery disease and/or aortic disease, which were finally excluded. The clinical characteristics of the study population and the control group are presented in Table 1.

**Table 1:** Baseline characteristics of AF patients and CG

	AF (*n* = 224)	CG (*n* = 40)
Age (years)	59 ± 9	47 ± 8
Gender (M/F)	129/95	16/15
Hypertension	140 (63%)	17 (53%)
BMI > 25	152 (68%)	14 (45%)
DM	23 (10%)	0
MI	15 (7%)	0
Stroke	11 (5%)	0
LVEF ECHO (%)	54 ± 9	60 ± 5

*MI*, myocardial infarction, *DM* diabetes mellitus, *BMI* body mas index

Seven patients were excluded from the study due to rare anomalies that are described in the results.

### MSCT of PVs

Multislice computed tomography (MSCT) scans were performed in all patients with a 64-slice Toshiba Multislice Aquilion System (Toshiba Medical System, Japan). Retrospective electrocardiographic gating was performed to minimize cardiac motion artifacts.

Non-ionic contrast material (Ultravist 370, Schering AG) was injected in the antebrachial vein (120 ml in three phases, phase one-70 ml, flow 5.0 ml/s-100% contrast, phase two- 30 ml the same flow-60% contrast and 40% saline, phase three-20 ml, 4.0 ml/s flow-100% saline, by means of a dual-head power injector-Injector CT2, Medtron, Germany).

Data sets were subsequently analyzed on Vitrea post-processing workstation (Vital Images) using 2-D and 3-D viewing modes. Electrocardiographically gated datasets were reconstructed automatically at different time of the R-R cycle length to approximate end-diastole phase of the cardiac cycle. Additional reconstruction windows were constructed after the examination of datasets if motion artifacts were present. Scans were analyzed by consensus of two observers. Images were evaluated using 0.5 mm thin-slab maximum intensity projections (MIP) and curved multiplanar reconstructions (cMPR).

LA diameters were measured with the maximal anterior-posterior distance in the oblique-sagittal view. The PV anatomy was assessed - the number of PV, common ostia and the branching pattern. The diameters of PV ostia were measured in two directions (anterior-posterior (AP) and superior-inferior (SI)). We defined the anomalies as follows in accordance with previous publications [3, 6, 8]:

- Additional pulmonary vein (APV) was defined as an extra PV up to pattern of 4 PVs.
- Common ostia (CO) was defined when the distance between the virtual border of the LA and the bifurcation of both PVs was 0.5 cm or less.
- Early branching (EB) was specified as the bifurcation of the PV within 1 cm of origin from the LA.
- Venous ostium index (VOI) determined the ovality shape of ostia and was calculated by dividing MSCT measurements in the AP and the SI directions. When the ratio approaches 1.0 the ostium is more rounded, when it deviates from 1.0 the shape is more oval.

Endoluminal views were routinely rendered and allowed a precise visualisation of the pulmonary vein ostia, pulmonary vein orientation, distance to the first branch, geometry of pulmonary vein branches and common ostia.

## Statistical analysis

The baseline clinical parameters and the results of parametric data were compared using the two-sample t-tests for normally distributed continuous variables between AF and CG group (Student’s t-test or Wilcoxon matched paired test). The Chi-square test was used to analyze nonparametric data. All of the text and table results are expressed as means ± standard deviation (SD) from the mean or a number (percentage). Statistical evaluation was performed using the software Statistica ver. 8.0 Stat Soft Pl. *P* value < 0.05 was considered statistically significant.

## Results

### LA dimension

The mean value (measured in CT) of the LA size was significantly larger in AF group vs CG group (41.2 ± 6 mm vs 35.4 ± 4 mm, *p* < 0.0001). Twenty six (11.6%) of AF pts. had LA larger than 50 mm, in comparison to CG, were no pts. had LA so enlarged.

### Anatomical anomalies

All data are presented in Table 2. The typical anatomical pattern of the pulmonary vein described as two right and two left PVs was observed more often in CG than in AF group – 34 pts. (85%) vs 150 pts. (67%) pts.; *p* < 0.05.

**Table 2:** Results: comparison of anatomical anomalies in AF patients and CG pts.

		AF (n = 224)	CG (n = 40)	p
LA CT (mm)		41.2 ± 6	35.4 ± 4	< 0.0001
Additional PV	right	42 (18.7%)	0	< 0.05
	left	4 (1.8%)	0	0.45
Common ostium	right	5 (2%)	2 (6.4%)	0.13
	left	21 (9%)	4 (12,9%)	0.5
Other anomalies		6 (2.7%)	0	

PV anatomy anomalies – additional pulmonary veins, common ostia or early branching were found more frequently in the AF group – 83 pts. (37%) as compared to the CG – 6 pts. (15%); p < 0.05.

Forty two (18.7%) pts. from AF group had additional right PV (APV), contrary to the CG where no APVs were observed. The mean diameter of APV’s was significantly smaller than the mean diameter of main veins (AP 7.2 ± 1.6 mm, SI 7.3 ± 1.8 mm vs AP 14.7 ± 3.0 mm, SI 17.4 ± 2.4 mm, *p* < 0.0001).

We recognized two types of common ostia of PVs:

- a long trunk CO with more than 20 mm distance between the left atrium and the bifurcation (5 AF pts., 0 CG pts)
- a short trunk CO – less than 20 mm distance between the left atrium and the bifurcation (21 AF pts., 6 CG pts).

The mean size of common ostia in AF patients was significantly larger than the diameter of main veins (AP 16.7 ± 4.2 mm, SI 24.5 ± 5.5 mm, p < 0.0001). There was a trend towards left-sided single ostia especially in AF patients.

There was no difference in the occurrence of early branching (EB) in AF - 5% (11 pts) as compared to CG - 5% (2 pts).

**Fig. 1:**
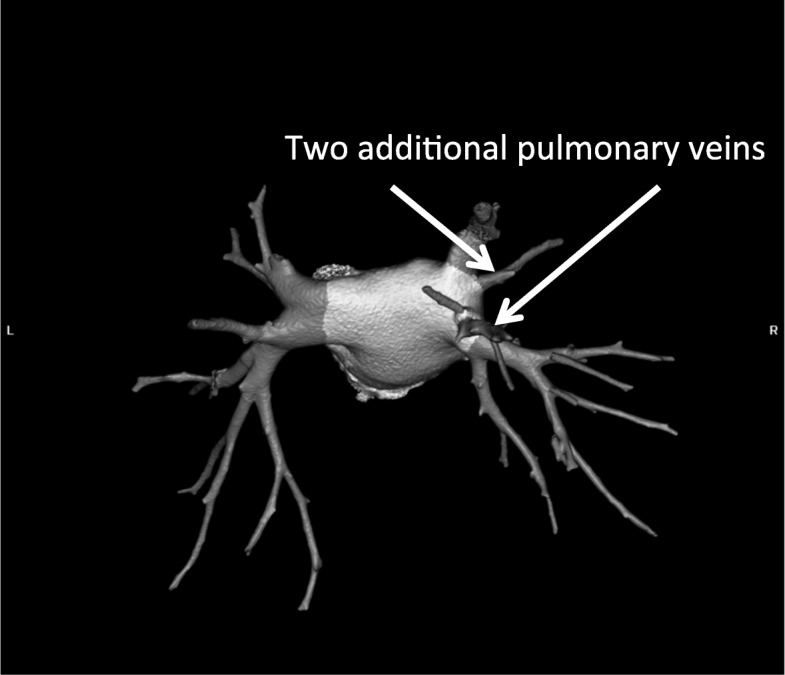
Computed tomography scan with three-dimensional reconstruction of pulmonary veins and the left atrium. The arrow indicates an additional pulmonary vein

**Fig. 2:**
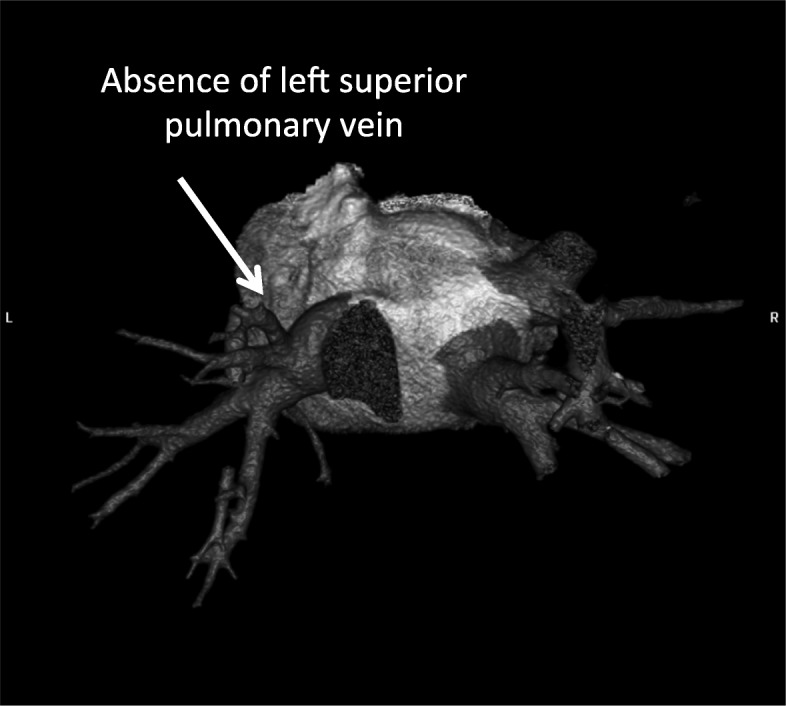
Three-dimensional reconstruction of pulmonary veins and left atrium. Arrow indicates a common truncus of the left pulmonary vein

**Fig. 3:**
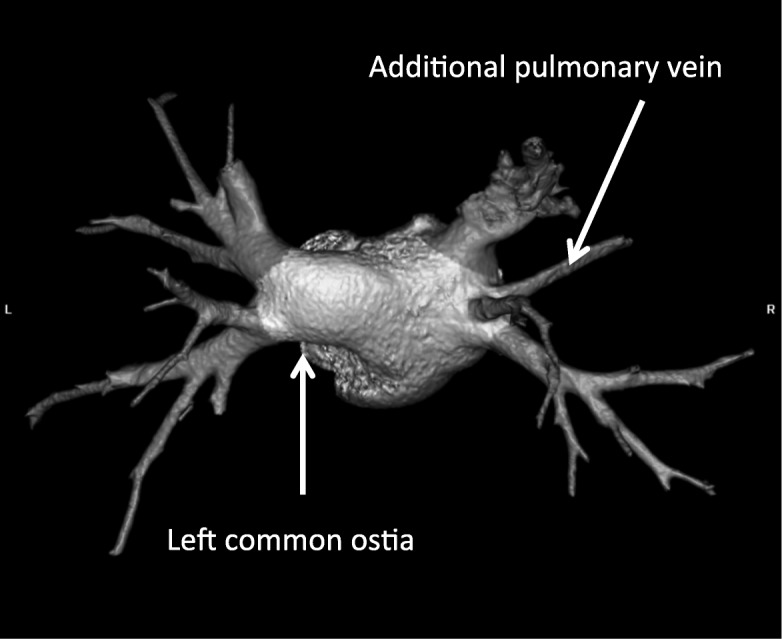
Left common ostia with right additional PV (middle PV)

**Fig. 4:**
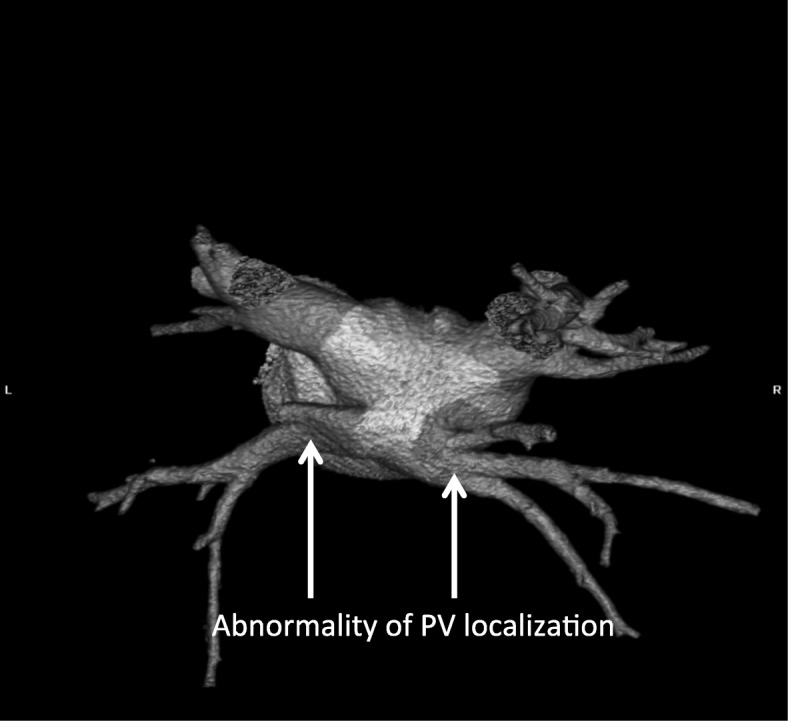
Abnormality of PV localization: all (4) PVs left were close to each other on the posterior wall of LA

In the study group some other anomalies were discovered:

- two additional PVs (right sided) - 1 pt. (Fig. 1);
- two additional PVs on the left and right side – 1 pt.;
- single left PV – 1 pt. (Fig. 2);
- and left common ostia with a right additional PV (middle PV) - in 2 pts. (Fig. 3);
- abnormality of PV localization: all four left PVs were close to each other on the backside of LA (Fig. 4).

### Pulmonary vein ostia

The AP and SI diameters of the PV ostia were significantly larger in the atrial fibrillation group than in the control group - results are shown in Table 3. In both groups the mean ostial diameter of the superior PV was larger than that of the inferior PV.

**Table 3:** Comparison results of SI and AP diameters of typical pattern 4 PVs in AF patients and CG (mean ± SD)

Pulmonary veins	AF (*n* = 224)	CG (*n* = 40)	p
*SI in mm*			
RSPV	18.5 ± 2.0	14.8 ± 2.9	< 0.0001
RIPV	16.8 ± 1.9	13.9 ± 3.4	< 0.0001
LSPV	18.2 ± 2.6	15.1 ± 2.3	< 0.0001
LIPV	16.1 ± 3.1	13.7 ± 2.7	< 0.0001
*AP in mm*			
RSPV	16.5 ± 3.0	13.7 ± 2.9	< 0.0001
RIPV	15.2 ± 2.1	13.6 ± 3.4	< 0.0001
LSPV	14.7 ± 2.7	12.0 ± 2.5	< 0.0001
LIPV	12.4 ± 4.3	10.3 ± 3.7	< 0.001

*RSPV* right superior pulmonary vein, *RIPV* right inferior pulmonary vein, *LSPV* left superior pulmonary vein, *LIPV* left inferior pulmonary vein

AF (+): right superior vs. right inferior PV - SI *p* < 0.0001, AP *p* < 0.02

left superior vs. left inferior PV - SI p < 0.0001, AP *p* < 0.0001,

GC: right superior vs. right inferior PV - SI *p* < 0.005, AP NS,

left superior vs left inferior PV - SI *p* < 0.05, AP p < 0.05

The venous ostium index (VOI) calculated for each PV proves the oval shape of most of them. Only the VOI of the right inferior pulmonary vein (RIPV) did differ between the groups (*p* < 0.005). Table 4 presents the VOI ratios.

**Table 4:** VOI ratio in AF patients and CG (mean ± SD)

VOI	AF *(n = 224)*	CG *(n = 40)*	p
RSPV	0.93 ± 0.2	0.94 ± 0.2	NS
RIPV	0.91 ± 0.1	1.02 ± 0.2	< 0.005
LSPV	0.83 ± 0.2	0.81 ± 0.2	NS
LIPV	0.79 ± 0.2	0.75 ± 0.2	NS

*RSPV* right superior pulmonary vein, *RIPV* right inferior pulmonary vein, *LSPV* left superior pulmonary vein, *LIPV* left inferior pulmonary vein

### Incidental findings

We also found rare anomalies in 7 pts. with AF – they were excluded from the analysis but we find it worth presenting. Partially anomalous pulmonary venous return was observed in 2 pts., atrial septal defect with anomalous insertion right upper PV into the right atrium - in 1 pt., and anomalous insertion of the middle meningeal vein into the right upper pulmonary vein - in 1 pt. One patient had right atrial diverticulum and 1 pt. left atrial diverticulum with a clot. No other clots were found in the LA. Right atrial myxoma localised in the interatrialis septum was diagnosed in 1 pt.

## Discussion

Several researchers, cardiologists, radiologists and surgeons studied the anatomy of LA and PV because of a variety of endovascular and surgical techniques used for invasive therapy of patients with AF [2–7].

This study once again proves the existence of significant differences in anatomy of LA and PV in AF patients in comparison with healthy individuals [2, 3]. This study presents a significantly larger group of pts. (224 pts) with AF than our previous publication [8] (82 pts) and other previously published papers – Bittner et al. [9] (166 pts. with AF.), Kubala et al. [7] 118 pts. with AF. Also the number of PV anomalies is greater than in other studies. [7–10]. In reference to Chen (710 pts. with AF, 710 pts. CG) et al. [11] our study is consistent with the dimensions of PVs - larger PVs in AF group, in contrast to our findings in Chen’s study the occurrence of PV variants did not differ between the groups. On the other hand one of the most extensive studies describing PV anatomy by Tekbas et al. shows the prevalence of PV abnormalities only in 26 (3,3%) out of 783 patients without atrial fibrillation [12].

The VOI ratio defining the shape of the pulmonary vein ostium is useful information for the operator - some research suggest it may define the method of ablatoin (cry ablation vs radio-frequency), predict the outcome and the complications (such as PV occlusion) [13].

Prior studies have proven that MSCT and cardiac magnetic resonance (CMR) are appropriate, non-invasive, widely available tools for describing LA anatomy and PV attachments in the patients qualified for the ablation procedure [2–16]. Moreover, CMR images allow for non-invasive therapy stratification using the atrial wall tissue characterization map for fibrosis assessment. It seems to be a good predictor for procedural outcome [2–10]. Although it has to be noticed that nowadays the left atrial model reconstruction with imaging by the electroanatomical mapping system is more often performed before the ablation procedure and CT/CMR imaging is not necessary to achieve good results and/or minimize complications [17, 18]. Thus it doesn’t change the significance of our findings.

Similarly to previous observations [2, 3, 8, 19–21] the data collected confirm larger diameters of LA, superior and inferior PVs in patients with AF. We want this study to underline the complexity of anatomical changes that may trigger AF.

It has been suggested that those anomalies may affect the results of pulmonary vein ablation [7, 8, 22–24]. Kubala et al. describes a superior clinical outcome in patients with typical 4 PV pattern in comparison with the patients with the left CO [7]. Sohns et al. analyzed 138 pts. and proved the impact of PV anatomy on AF recurrence after PVI [24]. Mulder’s et al. study was not conclusive - he presented only statistical trends of the relation between PV anatomy and PVI efficacy [10].

In contrast, a few studies state the opposite [25], eg. a recent paper by Heeger et al. analizes the outcome of PVI using cryoballoon in 74 pts. with left common ostia PV; he found the efficacy of PVI equal in the study and the control group, although in some cases the presence of left CO of PV required a different PVI technique [26].

The lack of decisive opinion on PV anatomy calls for carrying out more studies with larger groups. The full recognition of AF pathophysiology, LA and PV anatomy and remodelling are essential for optimalization of AF treatment strategies.

It should be noted that rare pathological findings were observed in pts. with AF initially qualified for PVI. The precise left atrium visualization helped us to properly qualify the patient for ablation and avoid procedural complications.

Anatomical anomalies of LA and PV may potentially increase the periprocedural complications and affect PVI efficacy [27]. We consider identifying anatomy anomalies as an important factor that may change the operator’s approach and modify the therapeutic strategy.

The main limitation of this paper is the number of patients in the control group.

## Conclusions

The anomalies of pulmonary vein anatomy occur more often in pts. with atrial fibrillation. They can be defined as an image biomarkers of atrial fibrillation. The right additional pulmonary vein was the most common anomaly detected in AF patients as well as enlarged diameters of the LA and PV ostia. The differences of pulmonary veins anatomy apply not only to the number of PVs but also to the localization and shape of PV’s ostia.

## Abbreviations

AF: Atrial fibrillation
APV: Additional pulmonary vein
CG: Control group
CMR: Cardiac magnetic resonanse
CO: Common ostia
EB: Early branching
ECG: Electrocardiogram
MSCT: Multi-slice computer tomography
PTS: Patients
PVI: Pulmonary vein isolation
SD: Standart deviation
VOI: Venous ostium index
